# 3β-Hydroxy­friedelan-17β-carboxylic acid

**DOI:** 10.1107/S1600536808010295

**Published:** 2008-04-23

**Authors:** Hai-Yan Chen, Cui-Wu Lin, Guang-Ying Chen, Guang-Chuan Ou

**Affiliations:** a College of Chemistry and Chemical Engineering, Guangxi University, Nanning 530004, People’s Republic of China; b Department of Chemistry, Hainan Normal University, Haikou 571158, People’s Republic of China; c Department of Biology and Chemistry, Hunan University of Science and Engineering, Yongzhou 425100, People’s Republic of China

## Abstract

The title compound, C_30_H_50_O_3_, which was isolated from a marine endophytic fungus, is a new friedelan derivative. The mol­ecule contains five six-membered rings, which exhibit boat (ring *A*), distorted boat (ring *B*) and chair (rings *C*–*E*) conformations. The crystal structure is stabilized by inter­molecular O—H⋯O hydrogen bonds, which link neighbouring mol­ecules into 12-membered rings.

## Related literature

For general background, see: Chen *et al.* (2003 ▶, 2005 ▶, 2006*a* ▶,*b* ▶); Lin *et al.* (2001*a* ▶,*b* ▶, 2002 ▶). For related structures, see: Dhaneshwar *et al.* (1987 ▶); Fun *et al.* (2007 ▶); Mo (1977 ▶); Mo *et al.* (1989 ▶); Sun *et al.* (2004 ▶).
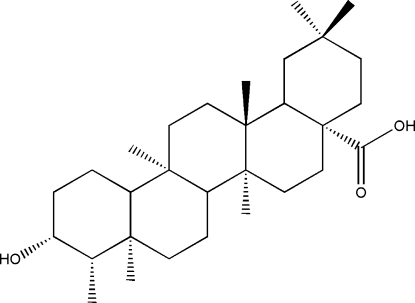


## Experimental

### 

#### Crystal data

C_30_H_50_O_3_
*M* *_r_* = 458.70
Orthorhombic, 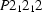

*a* = 13.238 (3) Å
*b* = 24.141 (5) Å
*c* = 8.7349 (17) Å
*V* = 2791.5 (10) Å^3^
*Z* = 4
Mo *K*α radiation
μ = 0.07 mm^−1^
*T* = 153 (2) K
0.51 × 0.48 × 0.46 mm

#### Data collection

Bruker SMART CCD area-detector diffractometer
Absorption correction: none
18448 measured reflections
3466 independent reflections
3010 reflections with *I* > 2σ(*I*)
*R*_int_ = 0.029

#### Refinement

*R*[*F* ^2^ > 2σ(*F* ^2^)] = 0.042
*wR*(*F* ^2^) = 0.112
*S* = 1.04
3466 reflections
308 parameters
H-atom parameters constrained
Δρ_max_ = 0.20 e Å^−3^
Δρ_min_ = −0.15 e Å^−3^

### 

Data collection: *SMART* (Bruker, 1998 ▶); cell refinement: *SAINT* (Bruker, 1998 ▶); data reduction: *SAINT*; program(s) used to solve structure: *SHELXTL* (Sheldrick, 2008 ▶); program(s) used to refine structure: *SHELXTL*; molecular graphics: *SHELXTL*; software used to prepare material for publication: *SHELXTL*.

## Supplementary Material

Crystal structure: contains datablocks global, I. DOI: 10.1107/S1600536808010295/xu2405sup1.cif

Structure factors: contains datablocks I. DOI: 10.1107/S1600536808010295/xu2405Isup2.hkl

Additional supplementary materials:  crystallographic information; 3D view; checkCIF report

## Figures and Tables

**Table 1:** Hydrogen-bond geometry (Å, °)

*D*—H⋯*A*	*D*—H	H⋯*A*	*D*⋯*A*	*D*—H⋯*A*
O35—H35⋯O41^i^	0.84	1.83	2.644 (2)	163
O41—H41⋯O34^ii^	0.84	1.89	2.721 (2)	168

Symmetry codes: (i) 
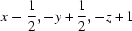
; (ii) 
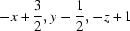
.

**Fig. 1.**
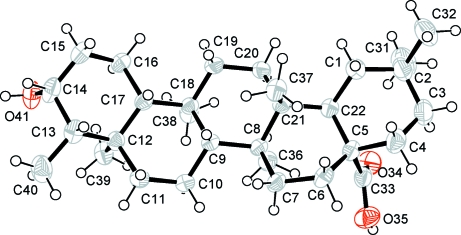
View of the title compound without the hydrogen atom-labeling scheme. Displacement ellipsoids are drawn at the 50% probability level.

**Fig. 2.**
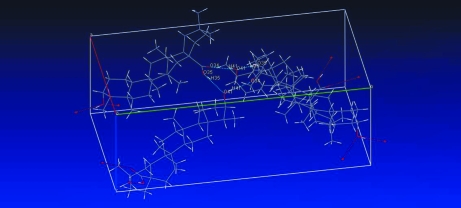
The unit cell packing of the title compound, showing the intermolecular hydrogen bonding completing a 12-membered ring.

## supplementary crystallographic information

### Comment

In the course of our ongoing research on the bioactive secondary metabolites from marine mangrove fungi (Chen *et al.*,  2003, 2005, 2006a, 2006b; Lin *et al.*, 2001*a*,b, 2002), the title compound was recently isolated from an unidentified species mangrove endophytic fungus No. H2K. It is a new friedelan analogue (Dhaneshwar *et al.*,  1987; Fun *et al.*,  2007; Mo, 1977; Mo *et al.*,  1989; Sun *et al.*,  2004). We report here the structure of the title compound. Further studies on properties are in progress.

The molecular structure is shown in Fig. 1. The molecule  contains five six-membered rins, they exhibit boat (ring A), distorted boat (ring B) and chair conformations (rings C–E ), respectively. The crystal structure is stabilized by intermolecular O—H···O hydrogen bonds, which link neighbouring molecules into 12-membered rings (Table 1 and Fig. 2).

### Experimental

Starter cultures were maintained on cornmeal seawater agar. Plugs of agar supporting mycelial growth were cut and transferred aseptically to a 250 ml Erlenmeyer flask containing 100 ml of liquid medium GYP (glucose 5 g/l, yeast extract 0.5 g/l, peptone 1 g/l,  beef extract 0.5 g/l, NaCl 3 g/l). The flask was incubated at 30°C on a rotary shaker for 5 d, and the mycelium was aseptically transferred to a 500 ml Erlenmeyer flask containing culture liquid (200 ml). The flask was then incubated at 30°C for 4 weeks. The cultures (80 l) were filtered through cheesecloth. The filtrate was concentrated to 5 l in vacuo below 50°C and extracted three times by shaking with an equal volume of ethyl acetate. The combined organic extracts were applied to a silica gel column, eluting with a gradient of petroleum ether to ethyl acetate to offer title compound (30 mg). The single crystals of the title compound were obtained by recrystallizing from an ethyl acetate/petroleum ether solution (V/V 1:1).

### Refinement

The H atoms placed in calculated positions with O—H = 0.84 Å and C–H = 0.98–1.00 Å, and refined in riding mode with *U*_iso_(H) = 1.5*U*_eq_(O,C) for hydroxyl groups and methyl groups, or U_iso_(H)= 1.2U_eq_(C) for others. As no significant anomalous scattering effect, Friedel pairs were merged.

### Crystal data

**Table:**

C_30_H_50_O_3_	*F*_000_ = 1016
*M**_r_* = 458.70	*D*_x_ = 1.092 Mg m^−^^3^
Orthorhombic, *P*2_1_2_1_2	Mo *K*α radiation λ = 0.71073 Å
Hall symbol: P 2 2ab	Cell parameters from 999 reflections
*a* = 13.238 (3) Å	θ = 3.0–26.8º
*b* = 24.141 (5) Å	µ = 0.07 mm^−^^1^
*c* = 8.7349 (17) Å	*T* = 153 (2) K
*V* = 2791.5 (10) Å^3^	Block, colourless
*Z* = 4	0.51 × 0.48 × 0.46 mm

### Data collection

**Table:**

Bruker SMART CCD area-detector diffractometer	3010 reflections with *I* > 2σ(*I*)
Radiation source: fine-focus sealed tube	*R*_int_ = 0.029
Monochromator: graphite	θ_max_ = 27.1º
*T* = 153(2) K	θ_min_ = 2.3º
ω scans	*h* = −16→16
Absorption correction: none	*k* = −23→30
18448 measured reflections	*l* = −9→11
3466 independent reflections	

### Refinement

**Table:**

Refinement on *F*^2^	Hydrogen site location: inferred from neighbouring sites
Least-squares matrix: full	H-atom parameters constrained
*R*[*F*^2^ > 2σ(*F*^2^)] = 0.042	*w* = 1/[σ^2^(*F*_o_^2^) + (0.0609*P*)^2^ + 0.5096*P*] where *P* = (*F*_o_^2^ + 2*F*_c_^2^)/3
*wR*(*F*^2^) = 0.112	(Δ/σ)_max_ < 0.001
*S* = 1.04	Δρ_max_ = 0.20 e Å^−^^3^
3457 reflections	Δρ_min_ = −0.15 e Å^−^^3^
308 parameters	Extinction correction: SHELXTL (Sheldrick, 2008), Fc^*^=kFc[1+0.001xFc^2^λ^3^/sin(2θ)]^-1/4^
Primary atom site location: structure-invariant direct methods	Extinction coefficient: 0.0119 (15)
Secondary atom site location: difference Fourier map	

### Special details

**Table:**

Geometry. All e.s.d.'s (except the e.s.d. in the dihedral angle between two l.s. planes) are estimated using the full covariance matrix. The cell e.s.d.'s are taken into account individually in the estimation of e.s.d.'s in distances, angles and torsion angles; correlations between e.s.d.'s in cell parameters are only used when they are defined by crystal symmetry. An approximate (isotropic) treatment of cell e.s.d.'s is used for estimating e.s.d.'s involving l.s. planes.
Refinement. Refinement of *F*^2^ against ALL reflections. The weighted *R*-factor *wR* and goodness of fit *S* are based on *F*^2^, conventional *R*-factors *R* are based on *F*, with *F* set to zero for negative *F*^2^. The threshold expression of *F*^2^ > σ(*F*^2^) is used only for calculating *R*-factors(gt) *etc*. and is not relevant to the choice of reflections for refinement. In absence of significant anomalous dispersion effects, Friedel-pair reflections were merged prior to refinement.*R*-factors based on *F*^2^ are statistically about twice as large as those based on *F*, and *R*- factors based on ALL data will be even larger.

### Fractional atomic coordinates and isotropic or equivalent isotropic displacement parameters (Å^2^)

**Table:**

	*x*	*y*	*z*	*U*_iso_*/*U*_eq_	
C1	0.81598 (18)	0.35284 (10)	−0.0686 (3)	0.0481 (6)	
H1A	0.8073	0.3249	−0.1506	0.058*	
H1B	0.8849	0.3483	−0.0266	0.058*	
C2	0.8082 (2)	0.41090 (11)	−0.1410 (3)	0.0556 (6)	
C3	0.6970 (2)	0.42285 (12)	−0.1844 (3)	0.0613 (7)	
H3A	0.6717	0.4537	−0.1203	0.074*	
H3B	0.6942	0.4350	−0.2926	0.074*	
C4	0.6285 (2)	0.37339 (11)	−0.1639 (3)	0.0529 (6)	
H4A	0.6502	0.3438	−0.2351	0.063*	
H4B	0.5588	0.3842	−0.1920	0.063*	
C5	0.62811 (16)	0.34982 (9)	0.0025 (2)	0.0373 (4)	
C6	0.56513 (16)	0.29657 (9)	0.0022 (3)	0.0421 (5)	
H6A	0.5873	0.2735	−0.0853	0.051*	
H6B	0.4937	0.3067	−0.0161	0.051*	
C7	0.57009 (16)	0.26112 (9)	0.1476 (3)	0.0427 (5)	
H7A	0.5629	0.2217	0.1180	0.051*	
H7B	0.5117	0.2707	0.2132	0.051*	
C8	0.66815 (14)	0.26743 (8)	0.2439 (2)	0.0321 (4)	
C9	0.69014 (13)	0.21204 (8)	0.3279 (2)	0.0319 (4)	
H9	0.6958	0.1841	0.2439	0.038*	
C10	0.60437 (15)	0.19038 (9)	0.4292 (3)	0.0425 (5)	
H10A	0.6020	0.2120	0.5254	0.051*	
H10B	0.5391	0.1952	0.3756	0.051*	
C11	0.62009 (16)	0.12897 (9)	0.4665 (3)	0.0440 (5)	
H11A	0.6162	0.1073	0.3704	0.053*	
H11B	0.5645	0.1164	0.5338	0.053*	
C12	0.72162 (15)	0.11659 (9)	0.5455 (2)	0.0367 (5)	
C13	0.74102 (18)	0.05269 (9)	0.5379 (3)	0.0447 (5)	
H13	0.7356	0.0428	0.4270	0.054*	
C14	0.84770 (19)	0.03498 (10)	0.5858 (3)	0.0508 (6)	
H14	0.8565	−0.0048	0.5568	0.061*	
C15	0.92836 (19)	0.06796 (11)	0.5046 (4)	0.0587 (7)	
H15A	0.9301	0.0569	0.3954	0.070*	
H15B	0.9949	0.0590	0.5499	0.070*	
C16	0.91100 (16)	0.13007 (10)	0.5149 (3)	0.0452 (5)	
H16A	0.9638	0.1497	0.4557	0.054*	
H16B	0.9160	0.1421	0.6230	0.054*	
C17	0.80690 (15)	0.14506 (9)	0.4519 (2)	0.0346 (4)	
H17	0.8043	0.1265	0.3497	0.042*	
C18	0.79289 (14)	0.20805 (8)	0.4146 (2)	0.0321 (4)	
C19	0.87692 (15)	0.22646 (9)	0.3048 (3)	0.0396 (5)	
H19A	0.9408	0.2297	0.3632	0.048*	
H19B	0.8866	0.1973	0.2265	0.048*	
C20	0.85661 (15)	0.28143 (9)	0.2237 (3)	0.0382 (5)	
H20A	0.9133	0.2895	0.1533	0.046*	
H20B	0.8540	0.3114	0.3009	0.046*	
C21	0.75695 (14)	0.28132 (8)	0.1319 (2)	0.0323 (4)	
C22	0.73960 (15)	0.34027 (8)	0.0593 (2)	0.0357 (4)	
H22	0.7521	0.3679	0.1424	0.043*	
C31	0.8474 (3)	0.45484 (13)	−0.0312 (4)	0.0836 (10)	
H31A	0.9188	0.4477	−0.0086	0.125*	
H31B	0.8083	0.4536	0.0640	0.125*	
H31C	0.8405	0.4915	−0.0782	0.125*	
C32	0.8739 (3)	0.41075 (15)	−0.2853 (4)	0.0878 (11)	
H32A	0.8739	0.4478	−0.3311	0.132*	
H32B	0.8466	0.3840	−0.3590	0.132*	
H32C	0.9432	0.4003	−0.2583	0.132*	
C33	0.57641 (18)	0.39522 (9)	0.0954 (3)	0.0428 (5)	
O34	0.61970 (15)	0.43196 (7)	0.1613 (2)	0.0584 (5)	
O35	0.47665 (13)	0.39178 (8)	0.0915 (2)	0.0593 (5)	
H35	0.4516	0.4172	0.1452	0.089*	
C36	0.65061 (17)	0.31401 (9)	0.3614 (3)	0.0424 (5)	
H36A	0.6119	0.2995	0.4482	0.064*	
H36B	0.6129	0.3442	0.3130	0.064*	
H36C	0.7159	0.3279	0.3977	0.064*	
C37	0.76840 (19)	0.23692 (9)	0.0047 (3)	0.0436 (5)	
H37A	0.8360	0.2395	−0.0404	0.065*	
H37B	0.7175	0.2432	−0.0748	0.065*	
H37C	0.7590	0.2000	0.0490	0.065*	
C38	0.80133 (19)	0.24406 (9)	0.5599 (2)	0.0439 (5)	
H38A	0.8499	0.2273	0.6307	0.066*	
H38B	0.7351	0.2465	0.6095	0.066*	
H38C	0.8244	0.2813	0.5319	0.066*	
C39	0.7150 (2)	0.13555 (10)	0.7141 (3)	0.0489 (6)	
H39A	0.6849	0.1726	0.7188	0.073*	
H39B	0.7829	0.1366	0.7585	0.073*	
H39C	0.6730	0.1095	0.7719	0.073*	
C40	0.6620 (2)	0.01711 (11)	0.6185 (4)	0.0617 (7)	
H40A	0.6789	−0.0221	0.6050	0.093*	
H40B	0.5954	0.0245	0.5743	0.093*	
H40C	0.6612	0.0261	0.7279	0.093*	
O41	0.86248 (15)	0.03944 (7)	0.7487 (2)	0.0578 (5)	
H41	0.8633	0.0076	0.7876	0.087*	

### Atomic displacement parameters (Å^2^)

**Table:**

	*U*^11^	*U*^22^	*U*^33^	*U*^12^	*U*^13^	*U*^23^
C1	0.0469 (12)	0.0492 (13)	0.0481 (13)	0.0055 (10)	0.0085 (11)	0.0088 (11)
C2	0.0647 (15)	0.0510 (14)	0.0512 (14)	0.0024 (12)	0.0137 (13)	0.0141 (12)
C3	0.0745 (17)	0.0560 (15)	0.0536 (15)	0.0137 (13)	0.0084 (14)	0.0184 (13)
C4	0.0593 (14)	0.0600 (15)	0.0392 (12)	0.0123 (12)	−0.0052 (11)	0.0072 (11)
C5	0.0429 (10)	0.0359 (10)	0.0330 (10)	0.0082 (9)	−0.0016 (9)	0.0002 (8)
C6	0.0422 (10)	0.0395 (11)	0.0447 (12)	0.0055 (9)	−0.0151 (10)	−0.0021 (10)
C7	0.0342 (10)	0.0398 (11)	0.0542 (13)	0.0007 (9)	−0.0101 (10)	0.0071 (11)
C8	0.0291 (9)	0.0343 (10)	0.0329 (10)	0.0038 (7)	−0.0019 (8)	0.0002 (8)
C9	0.0285 (8)	0.0340 (10)	0.0331 (10)	0.0019 (8)	−0.0017 (8)	−0.0014 (8)
C10	0.0308 (9)	0.0475 (12)	0.0493 (12)	0.0026 (9)	0.0001 (10)	0.0090 (10)
C11	0.0334 (9)	0.0456 (12)	0.0529 (13)	−0.0042 (9)	−0.0048 (10)	0.0085 (10)
C12	0.0369 (10)	0.0346 (10)	0.0388 (11)	−0.0008 (8)	−0.0062 (9)	0.0025 (9)
C13	0.0524 (12)	0.0369 (11)	0.0448 (13)	−0.0013 (10)	−0.0144 (11)	−0.0028 (10)
C14	0.0594 (14)	0.0329 (11)	0.0600 (15)	0.0073 (10)	−0.0166 (13)	0.0007 (10)
C15	0.0491 (13)	0.0558 (15)	0.0712 (17)	0.0179 (12)	−0.0058 (13)	0.0107 (14)
C16	0.0352 (10)	0.0495 (13)	0.0510 (13)	0.0043 (9)	−0.0082 (10)	0.0091 (11)
C17	0.0332 (9)	0.0382 (10)	0.0326 (10)	0.0043 (8)	−0.0049 (8)	−0.0025 (8)
C18	0.0297 (8)	0.0350 (10)	0.0317 (9)	0.0014 (8)	−0.0019 (8)	0.0002 (8)
C19	0.0285 (9)	0.0495 (12)	0.0409 (11)	0.0032 (8)	−0.0015 (9)	0.0053 (9)
C20	0.0312 (9)	0.0452 (12)	0.0383 (11)	−0.0017 (8)	0.0002 (9)	0.0048 (9)
C21	0.0326 (9)	0.0338 (10)	0.0305 (9)	0.0036 (8)	0.0001 (8)	−0.0020 (8)
C22	0.0389 (10)	0.0348 (10)	0.0333 (10)	0.0029 (8)	−0.0004 (9)	−0.0010 (8)
C31	0.101 (3)	0.0595 (18)	0.090 (2)	−0.0205 (18)	−0.002 (2)	0.0065 (18)
C32	0.101 (2)	0.083 (2)	0.079 (2)	0.018 (2)	0.043 (2)	0.0334 (19)
C33	0.0512 (12)	0.0364 (11)	0.0408 (11)	0.0091 (10)	0.0038 (10)	0.0049 (9)
O34	0.0668 (11)	0.0356 (8)	0.0727 (12)	0.0049 (8)	0.0050 (10)	−0.0131 (9)
O35	0.0503 (9)	0.0573 (11)	0.0702 (12)	0.0155 (8)	0.0074 (9)	−0.0078 (9)
C36	0.0477 (11)	0.0399 (11)	0.0397 (11)	0.0092 (9)	0.0083 (10)	−0.0004 (9)
C37	0.0546 (13)	0.0407 (12)	0.0353 (10)	0.0095 (10)	0.0019 (10)	−0.0049 (10)
C38	0.0547 (13)	0.0410 (11)	0.0359 (11)	−0.0025 (10)	−0.0086 (11)	−0.0019 (9)
C39	0.0626 (14)	0.0422 (12)	0.0419 (12)	−0.0001 (11)	0.0034 (11)	0.0026 (10)
C40	0.0683 (16)	0.0443 (13)	0.0725 (18)	−0.0146 (12)	−0.0183 (15)	0.0096 (13)
O41	0.0721 (11)	0.0384 (9)	0.0628 (11)	−0.0092 (8)	−0.0293 (10)	0.0121 (8)

### Geometric parameters (Å, °)

**Table:**

C1—C22	1.537 (3)	C15—H15A	0.9900
C1—C2	1.541 (3)	C15—H15B	0.9900
C1—H1A	0.9900	C16—C17	1.528 (3)
C1—H1B	0.9900	C16—H16A	0.9900
C2—C31	1.522 (4)	C16—H16B	0.9900
C2—C32	1.531 (4)	C17—C18	1.566 (3)
C2—C3	1.547 (4)	C17—H17	1.0000
C3—C4	1.510 (4)	C18—C19	1.535 (3)
C3—H3A	0.9900	C18—C38	1.542 (3)
C3—H3B	0.9900	C19—C20	1.528 (3)
C4—C5	1.561 (3)	C19—H19A	0.9900
C4—H4A	0.9900	C19—H19B	0.9900
C4—H4B	0.9900	C20—C21	1.544 (3)
C5—C33	1.526 (3)	C20—H20A	0.9900
C5—C6	1.532 (3)	C20—H20B	0.9900
C5—C22	1.574 (3)	C21—C37	1.551 (3)
C6—C7	1.533 (3)	C21—C22	1.575 (3)
C6—H6A	0.9900	C22—H22	1.0000
C6—H6B	0.9900	C31—H31A	0.9800
C7—C8	1.555 (3)	C31—H31B	0.9800
C7—H7A	0.9900	C31—H31C	0.9800
C7—H7B	0.9900	C32—H32A	0.9800
C8—C36	1.540 (3)	C32—H32B	0.9800
C8—C9	1.553 (3)	C32—H32C	0.9800
C8—C21	1.566 (3)	C33—O34	1.203 (3)
C9—C10	1.531 (3)	C33—O35	1.324 (3)
C9—C18	1.560 (3)	O35—H35	0.8400
C9—H9	1.0000	C36—H36A	0.9800
C10—C11	1.532 (3)	C36—H36B	0.9800
C10—H10A	0.9900	C36—H36C	0.9800
C10—H10B	0.9900	C37—H37A	0.9800
C11—C12	1.540 (3)	C37—H37B	0.9800
C11—H11A	0.9900	C37—H37C	0.9800
C11—H11B	0.9900	C38—H38A	0.9800
C12—C39	1.544 (3)	C38—H38B	0.9800
C12—C17	1.554 (3)	C38—H38C	0.9800
C12—C13	1.565 (3)	C39—H39A	0.9800
C13—C40	1.525 (4)	C39—H39B	0.9800
C13—C14	1.534 (3)	C39—H39C	0.9800
C13—H13	1.0000	C40—H40A	0.9800
C14—O41	1.440 (3)	C40—H40B	0.9800
C14—C15	1.509 (4)	C40—H40C	0.9800
C14—H14	1.0000	O41—H41	0.8400
C15—C16	1.520 (3)		
			
C22—C1—C2	115.72 (19)	C15—C16—C17	110.41 (19)
C22—C1—H1A	108.4	C15—C16—H16A	109.6
C2—C1—H1A	108.4	C17—C16—H16A	109.6
C22—C1—H1B	108.4	C15—C16—H16B	109.6
C2—C1—H1B	108.4	C17—C16—H16B	109.6
H1A—C1—H1B	107.4	H16A—C16—H16B	108.1
C31—C2—C32	109.1 (3)	C16—C17—C12	111.15 (17)
C31—C2—C1	110.6 (2)	C16—C17—C18	114.32 (17)
C32—C2—C1	107.3 (2)	C12—C17—C18	116.92 (16)
C31—C2—C3	110.4 (3)	C16—C17—H17	104.3
C32—C2—C3	109.8 (3)	C12—C17—H17	104.3
C1—C2—C3	109.5 (2)	C18—C17—H17	104.3
C4—C3—C2	113.3 (2)	C19—C18—C38	107.38 (17)
C4—C3—H3A	108.9	C19—C18—C9	108.09 (15)
C2—C3—H3A	108.9	C38—C18—C9	115.35 (17)
C4—C3—H3B	108.9	C19—C18—C17	108.98 (16)
C2—C3—H3B	108.9	C38—C18—C17	111.58 (17)
H3A—C3—H3B	107.7	C9—C18—C17	105.31 (15)
C3—C4—C5	113.6 (2)	C20—C19—C18	114.44 (17)
C3—C4—H4A	108.8	C20—C19—H19A	108.7
C5—C4—H4A	108.8	C18—C19—H19A	108.7
C3—C4—H4B	108.8	C20—C19—H19B	108.7
C5—C4—H4B	108.8	C18—C19—H19B	108.7
H4A—C4—H4B	107.7	H19A—C19—H19B	107.6
C33—C5—C6	111.08 (18)	C19—C20—C21	112.93 (17)
C33—C5—C4	103.59 (17)	C19—C20—H20A	109.0
C6—C5—C4	107.80 (18)	C21—C20—H20A	109.0
C33—C5—C22	111.00 (17)	C19—C20—H20B	109.0
C6—C5—C22	112.84 (16)	C21—C20—H20B	109.0
C4—C5—C22	110.08 (18)	H20A—C20—H20B	107.8
C5—C6—C7	116.32 (18)	C20—C21—C37	106.84 (16)
C5—C6—H6A	108.2	C20—C21—C8	108.48 (15)
C7—C6—H6A	108.2	C37—C21—C8	111.90 (17)
C5—C6—H6B	108.2	C20—C21—C22	109.41 (16)
C7—C6—H6B	108.2	C37—C21—C22	110.52 (16)
H6A—C6—H6B	107.4	C8—C21—C22	109.61 (15)
C6—C7—C8	115.44 (18)	C1—C22—C5	111.04 (18)
C6—C7—H7A	108.4	C1—C22—C21	112.03 (17)
C8—C7—H7A	108.4	C5—C22—C21	113.32 (16)
C6—C7—H7B	108.4	C1—C22—H22	106.7
C8—C7—H7B	108.4	C5—C22—H22	106.7
H7A—C7—H7B	107.5	C21—C22—H22	106.7
C36—C8—C9	110.03 (16)	C2—C31—H31A	109.5
C36—C8—C7	107.84 (16)	C2—C31—H31B	109.5
C9—C8—C7	109.14 (16)	H31A—C31—H31B	109.5
C36—C8—C21	111.92 (16)	C2—C31—H31C	109.5
C9—C8—C21	109.81 (15)	H31A—C31—H31C	109.5
C7—C8—C21	108.02 (16)	H31B—C31—H31C	109.5
C10—C9—C8	115.34 (16)	C2—C32—H32A	109.5
C10—C9—C18	110.17 (16)	C2—C32—H32B	109.5
C8—C9—C18	116.49 (15)	H32A—C32—H32B	109.5
C10—C9—H9	104.4	C2—C32—H32C	109.5
C8—C9—H9	104.4	H32A—C32—H32C	109.5
C18—C9—H9	104.4	H32B—C32—H32C	109.5
C11—C10—C9	110.65 (17)	O34—C33—O35	122.3 (2)
C11—C10—H10A	109.5	O34—C33—C5	124.8 (2)
C9—C10—H10A	109.5	O35—C33—C5	112.9 (2)
C11—C10—H10B	109.5	C33—O35—H35	109.5
C9—C10—H10B	109.5	C8—C36—H36A	109.5
H10A—C10—H10B	108.1	C8—C36—H36B	109.5
C10—C11—C12	113.68 (18)	H36A—C36—H36B	109.5
C10—C11—H11A	108.8	C8—C36—H36C	109.5
C12—C11—H11A	108.8	H36A—C36—H36C	109.5
C10—C11—H11B	108.8	H36B—C36—H36C	109.5
C12—C11—H11B	108.8	C21—C37—H37A	109.5
H11A—C11—H11B	107.7	C21—C37—H37B	109.5
C11—C12—C39	108.68 (19)	H37A—C37—H37B	109.5
C11—C12—C17	108.18 (17)	C21—C37—H37C	109.5
C39—C12—C17	114.32 (18)	H37A—C37—H37C	109.5
C11—C12—C13	108.38 (17)	H37B—C37—H37C	109.5
C39—C12—C13	109.99 (18)	C18—C38—H38A	109.5
C17—C12—C13	107.12 (17)	C18—C38—H38B	109.5
C40—C13—C14	110.4 (2)	H38A—C38—H38B	109.5
C40—C13—C12	115.0 (2)	C18—C38—H38C	109.5
C14—C13—C12	114.48 (18)	H38A—C38—H38C	109.5
C40—C13—H13	105.3	H38B—C38—H38C	109.5
C14—C13—H13	105.3	C12—C39—H39A	109.5
C12—C13—H13	105.3	C12—C39—H39B	109.5
O41—C14—C15	109.2 (2)	H39A—C39—H39B	109.5
O41—C14—C13	111.9 (2)	C12—C39—H39C	109.5
C15—C14—C13	112.1 (2)	H39A—C39—H39C	109.5
O41—C14—H14	107.8	H39B—C39—H39C	109.5
C15—C14—H14	107.8	C13—C40—H40A	109.5
C13—C14—H14	107.8	C13—C40—H40B	109.5
C14—C15—C16	112.7 (2)	H40A—C40—H40B	109.5
C14—C15—H15A	109.1	C13—C40—H40C	109.5
C16—C15—H15A	109.1	H40A—C40—H40C	109.5
C14—C15—H15B	109.1	H40B—C40—H40C	109.5
C16—C15—H15B	109.1	C14—O41—H41	109.5
H15A—C15—H15B	107.8		
			
C22—C1—C2—C31	72.8 (3)	C13—C12—C17—C18	168.25 (17)
C22—C1—C2—C32	−168.3 (3)	C10—C9—C18—C19	176.21 (18)
C22—C1—C2—C3	−49.1 (3)	C8—C9—C18—C19	−50.0 (2)
C31—C2—C3—C4	−129.2 (3)	C10—C9—C18—C38	−63.6 (2)
C32—C2—C3—C4	110.5 (3)	C8—C9—C18—C38	70.2 (2)
C1—C2—C3—C4	−7.1 (3)	C10—C9—C18—C17	59.8 (2)
C2—C3—C4—C5	57.6 (3)	C8—C9—C18—C17	−166.34 (16)
C3—C4—C5—C33	68.3 (3)	C16—C17—C18—C19	55.0 (2)
C3—C4—C5—C6	−174.0 (2)	C12—C17—C18—C19	−172.64 (16)
C3—C4—C5—C22	−50.5 (3)	C16—C17—C18—C38	−63.4 (2)
C33—C5—C6—C7	−77.6 (2)	C12—C17—C18—C38	69.0 (2)
C4—C5—C6—C7	169.56 (19)	C16—C17—C18—C9	170.78 (17)
C22—C5—C6—C7	47.8 (3)	C12—C17—C18—C9	−56.9 (2)
C5—C6—C7—C8	−25.9 (3)	C38—C18—C19—C20	−75.4 (2)
C6—C7—C8—C36	90.4 (2)	C9—C18—C19—C20	49.6 (2)
C6—C7—C8—C9	−150.07 (18)	C17—C18—C19—C20	163.59 (17)
C6—C7—C8—C21	−30.7 (2)	C18—C19—C20—C21	−57.0 (2)
C36—C8—C9—C10	62.4 (2)	C19—C20—C21—C37	−63.1 (2)
C7—C8—C9—C10	−55.8 (2)	C19—C20—C21—C8	57.7 (2)
C21—C8—C9—C10	−174.02 (17)	C19—C20—C21—C22	177.21 (17)
C36—C8—C9—C18	−69.1 (2)	C36—C8—C21—C20	67.5 (2)
C7—C8—C9—C18	172.75 (17)	C9—C8—C21—C20	−55.0 (2)
C21—C8—C9—C18	54.5 (2)	C7—C8—C21—C20	−173.94 (16)
C8—C9—C10—C11	162.92 (18)	C36—C8—C21—C37	−174.90 (17)
C18—C9—C10—C11	−62.7 (2)	C9—C8—C21—C37	62.6 (2)
C9—C10—C11—C12	57.3 (3)	C7—C8—C21—C37	−56.3 (2)
C10—C11—C12—C39	75.3 (2)	C36—C8—C21—C22	−51.9 (2)
C10—C11—C12—C17	−49.4 (2)	C9—C8—C21—C22	−174.43 (16)
C10—C11—C12—C13	−165.2 (2)	C7—C8—C21—C22	66.6 (2)
C11—C12—C13—C40	−61.1 (3)	C2—C1—C22—C5	55.1 (3)
C39—C12—C13—C40	57.6 (3)	C2—C1—C22—C21	−177.0 (2)
C17—C12—C13—C40	−177.61 (19)	C33—C5—C22—C1	−118.2 (2)
C11—C12—C13—C14	169.5 (2)	C6—C5—C22—C1	116.4 (2)
C39—C12—C13—C14	−71.8 (3)	C4—C5—C22—C1	−4.1 (2)
C17—C12—C13—C14	52.9 (3)	C33—C5—C22—C21	114.73 (19)
C40—C13—C14—O41	−58.8 (3)	C6—C5—C22—C21	−10.7 (2)
C12—C13—C14—O41	72.9 (3)	C4—C5—C22—C21	−131.17 (18)
C40—C13—C14—C15	178.1 (2)	C20—C21—C22—C1	69.3 (2)
C12—C13—C14—C15	−50.2 (3)	C37—C21—C22—C1	−48.1 (2)
O41—C14—C15—C16	−74.1 (3)	C8—C21—C22—C1	−171.89 (18)
C13—C14—C15—C16	50.5 (3)	C20—C21—C22—C5	−164.12 (17)
C14—C15—C16—C17	−56.4 (3)	C37—C21—C22—C5	78.5 (2)
C15—C16—C17—C12	61.2 (3)	C8—C21—C22—C5	−45.3 (2)
C15—C16—C17—C18	−163.8 (2)	C6—C5—C33—O34	151.5 (2)
C11—C12—C17—C16	−174.64 (19)	C4—C5—C33—O34	−93.0 (3)
C39—C12—C17—C16	64.1 (2)	C22—C5—C33—O34	25.1 (3)
C13—C12—C17—C16	−58.0 (2)	C6—C5—C33—O35	−31.2 (3)
C11—C12—C17—C18	51.6 (2)	C4—C5—C33—O35	84.3 (2)
C39—C12—C17—C18	−69.6 (2)	C22—C5—C33—O35	−157.59 (19)

### Hydrogen-bond geometry (Å, °)

**Table:**

*D*—H···*A*	*D*—H	H···*A*	*D*···*A*	*D*—H···*A*
O35—H35···O41^i^	0.84	1.83	2.644 (2)	163
O41—H41···O34^ii^	0.84	1.89	2.721 (2)	168

Symmetry codes: (i) *x*−1/2, −*y*+1/2, −*z*+1; (ii) −*x*+3/2, *y*−1/2, −*z*+1.
